# Recurrent trigeminocardiac reflex in percutaneous balloon compression for trigeminal neuralgia: A case report

**DOI:** 10.1097/MD.0000000000022467

**Published:** 2020-10-30

**Authors:** Qin Qin, Yaping Wang

**Affiliations:** a Department of Anesthesiology, Xiangya Second Hospital, Central South University, Changsha, Hunan; b Department of Anesthesiology, Wuxi Maternity and Child Health Hospital Affiliated to Nanjing Medical University, Wuxi, Jiangsu, China.

**Keywords:** isoproterenol, percutaneous balloon compression, trigeminal neuralgia, trigeminocardiac reflex

## Abstract

**Rationale:**

Trigeminocardiac reflex (TCR) sometimes occurs during the percutaneous balloon compression (PBC) procedure to treat trigeminal neuralgia (TN), and it manifests as transient bradycardia or sinus arrest. However, recurrent intraoperative TCR cases are rarely reported. Meanwhile, the treatment for recurrent TCR is still unclear.

**Patient concerns:**

A 74-year-old male with a 2-year TN history could no longer tolerate the side effects of carbamazepine and came to seek PBC treatment.

**Diagnoses:**

Bradycardia or sinus arrest occurred repeatedly during the operation, and the heart rate (HR) rapidly returned to normal when the operation was suspended. The C-arm image showed the puncture needle entering the foramen ovale.

**Interventions:**

First, 0.5 mg atropine was administered twice, and then 1 mL of 2% lidocaine was injected locally at the puncture site. Finally, isoproterenol was continuously pumped and dynamically adjusted to maintain the HR above 90 bpm.

**Outcomes:**

The use of atropine and lidocaine did not prevent the recurrence of TCR. The use of isoproterenol to maintain the HR enabled the successful completion of the operation. The patient recovered quickly after the operation and was discharged 2 days later. No complaints of discomfort were reported during the sixth-month follow-up.

**Lessons:**

The elimination of intraoperative TCR may be difficult. Maintaining a high HR intraoperatively by continuous isoproterenol infusion is effective for preventing or mitigating the onset of TCR.

## 1 Introduction

Percutaneous balloon compression (PBC) is widely used for the treatment of trigeminal neuralgia (TN).^[1]^ However, clinical observations have found that transient heart rate (HR) changes (bradycardia or even sinus arrest) often occur during PBC.^[2,3]^ This phenomenon, caused by stimulation of the fifth paired cranial nerve (trigeminal nerve), is called the trigeminocardiac reflex (TCR).^[4]^

Here, we present a case of recurrent TCR during PBC when the puncture needle entered the foramen ovale. The intravenous use of atropine and local injection of lidocaine at the puncture site failed to stop the occurrence of the next TCR. Finally, we maintained the HR at approximately 90 bpm via continuous infusion with isoproterenol and were able to complete the operation.

## 2 Case report

A 74-year-old male (weight: 66 kg, height: 168 cm) with a 2-year history of left TN was receiving regular carbamazepine (200 mg bid) treatment. However, his symptoms aggravated over the past year. The patient found it difficult to tolerate the side effects of larger doses of carbamazepine (dizziness) and came to seek PBC treatment. The patient had a history of hypertension, and blood pressure was controlled around 140/90 mm Hg under medication. Preoperative electrocardiogram (ECG), chest X-ray, and related laboratory examinations showed no abnormalities. A head magnetic resonance imaging (MRI) showed bilateral trigeminal vascular compression with a more obvious compression on the left side.

After the patient entered the operating room, 12-ECG, SpO2, bispectral index (BIS), and continuous arterial pressure monitoring (Mindray T9, China) were performed. Anesthesia was induced by intravenous injection of midazolam (4 mg), propofol (50 mg), sufentanil (25 μg), and vecuronium bromide (8 mg), with mechanical ventilation after intubation. Anesthesia was maintained by continuous infusion with propofol and remifentanil, maintaining the BIS between 40 and 50. All vital signs were stable after anesthesia.

**Figure 1:**
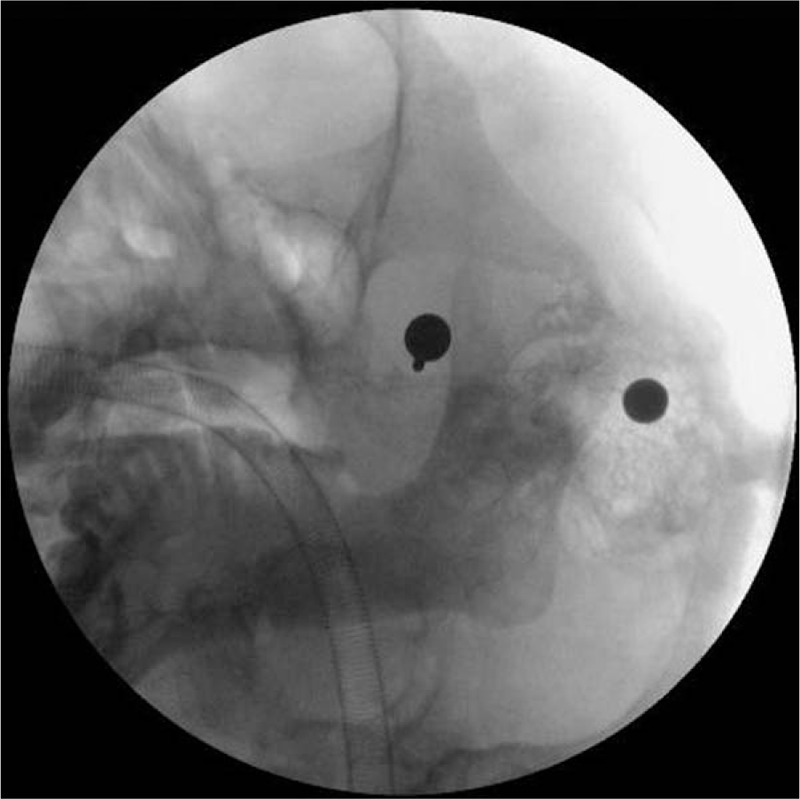
The C-arm fluoroscopy at the first occurrence of trigeminocardiac reflex.

**Figure 2:**
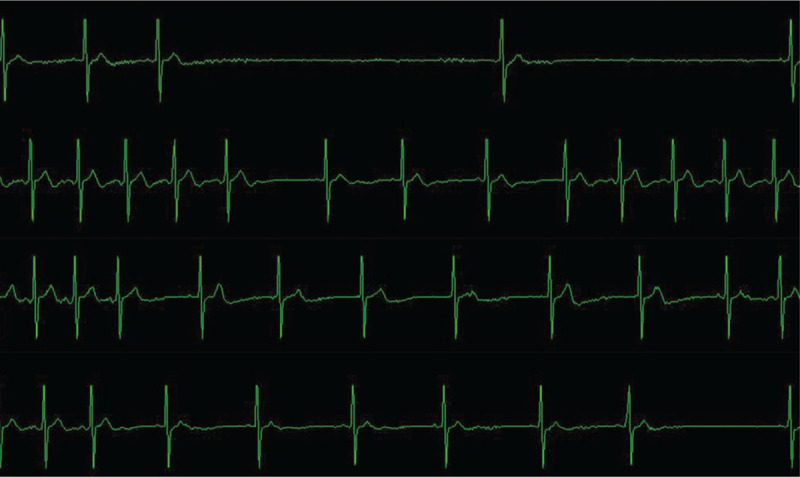
Electrocardiogram of the trigeminocardiac reflex happened after the use of atropine.

At the beginning of the operation, 0.5 mg atropine was administered. Sudden sinus arrest (lasting 5 seconds) occurred during puncture; the operation was stopped immediately, and HR quickly returned to normal. C-arm fluoroscopy showed the puncture needle entering the foramen ovale (Fig. 1). Due to safety considerations, we administered 0.5 mg atropine again. However, when we reoperated the puncture needle, bradycardia occurred (HR change: 80 to 47 bpm). The HR returned to normal after immediate cessation, while bradycardia recurred (HR changes: 89 to 43 bpm, 74 to 31 bpm) as soon as the puncture needle moved slightly (Fig. 2).

**Figure 3:**
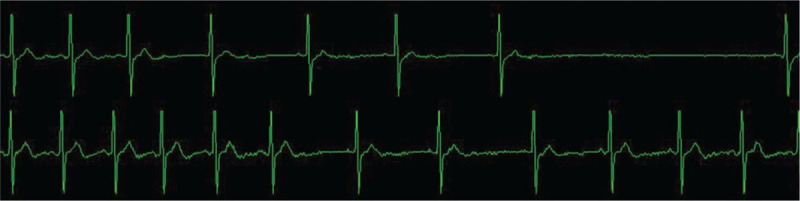
Electrocardiogram of the trigeminocardiac reflex happened after the local injection of lidocaine.

**Figure 4:**
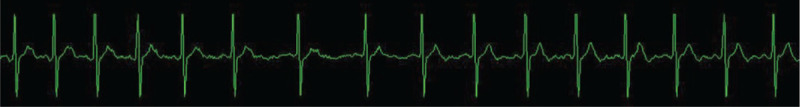
Electrocardiogram of a slight heart rate change happened after the continuous infusion of isoprenaline.

For the safety of the patient, we requested assistance from the superior doctor. After the arrival of the professor, 2% lidocaine (1 mL) was injected into the puncture site through the puncture needle. After 3 minutes, we started the operation again. However, transient sinus arrest reappeared (lasting 4 seconds), and the operation was suspended again. The was no improvement after another 2 minutes of waiting, and bradycardia occurred again (HR change: 77 to 49 bpm) (Fig. 3). Thus, we decided to try one last time before canceling the operation. Isoproterenol (1 mg + 25 mL normal saline) was continuously pumped to the patient, and the speed was dynamically adjusted to maintain the HR at approximately 90 bpm. Norepinephrine (2 mg) was administered when the blood pressure was below 90/60 mm Hg. We continued the operation again after stabilizing the HR, with only a slight decrease in HR (HR change: 89 to 72 bpm) (Fig. 4), and we completed the operation. All medications were discontinued upon cessation of the balloon compression. Intraoperative blood pressure was stable, and mean arterial pressure fluctuated between 60 and 102 mm Hg.

The patient recovered quickly after the operation and was clearly conscious with facial numbness (an indicator of the effectiveness of the operation). No complaints of discomfort were reported after the operation. The postoperative ECG was normal, and the patient was discharged satisfactorily. During follow-up period, the patient showed significant pain relief and no discomfort.

## 3 Discussion and conclusions

TCR is defined as the sudden onset of parasympathetic dysrhythmia, sympathetic hypotension, apnea, or hyperglycemia during the stimulation of any sensory branch of the trigeminal nerve, and the HR often changes over 20% of the baseline.^[4]^ Chowdhury et al^[5]^ classified TCR into 3 types; TCR during PBC belongs to the type that results from direct stimulation to the Gasserian ganglion, and it usually manifests as transient bradycardia or sinus arrest, with or without a dramatic increase in blood pressure. The incidence of TCR-related HR changes during PBC is very high. Although there is no specific literature on these statistics, it is about 50% to 80% according to relevant studies.^[2,3,6]^ Sudden changes in HR and blood pressure can be dangerous and even fatal to patients. Therefore, correct recognition and prompt treatment of this phenomenon are of great importance to the surgeons and anesthesiologists.

The precise mechanism of TCR occurrence is still not fully understood, but it is widely believed that when the surgical operation stimulates the trigeminal nerve branch or ganglion, it sends a stimulus to the trigeminal nerve sensory nucleus, which then transmits the stimulus to the parasympathetic neurons in the motor nucleus of the vagus nerve.^[7]^ Stimulation of the vagus nerve causes bradycardia and sinus arrest. Therefore, in this case, when the puncture needle entered the foramen ovale and when it was moved, bradycardia or sinus arrest (HR change over 20%) occurred, and the HR quickly returned to normal after suspending the operation. Thus, we diagnosed it as TCR.

In this case, minor operations led to recurrent bradycardia or even sinus arrest, which seriously affected the continuation of the operation. The prophylactic use of atropine has been proven to reduce intraoperative TCR.^[6,8]^ However, in this case, the prophylactic use of atropine at the beginning of the operation failed to prevent the occurrence of transient sinus arrest when the puncture needle entered the foramen ovale, and the subsequent use of atropine failed to prevent the recurrence of bradycardia. Dominguez et al^[3]^ and Tibano et al^[9]^ all attempted to perform a trigeminal nerve block (1–2% lidocaine, 0.8–1 mL) before surgery. The use of local anesthesia for the trigeminal ganglion was helpful to reduce the fluctuation of intraoperative HR. In addition, Chigurupati et al^[10]^ successfully used local lidocaine infiltration to complete a trigeminal microvascular decompression operation with a similar situation, where TCR recurred during the operation. However, in this case, the use of local anesthetics did not prevent sinus arrest or bradycardia induced by reoperation, and increased waiting time for infiltration was not effective. In the past, the use of a temporary pacemaker was considered for operation.^[2]^ However, in this case, although bradycardia and sinus arrest occurred frequently, the onset was transient and the condition returned to normal within a few seconds, so the use of pacemaker may not be suitable. Isoproterenol is a β-receptor agonist; when acting on the cardiac β1 receptor, it helps to strengthen the myocardial contractility and increase the HR.^[11]^ Although no relevant literature had used it during PBC surgery, the clinical characteristics of bradycardia during PBC surgery prompted us to consider continuously pumping isoproterenol to increase the HR during surgery. We did this to prevent and buffer the recurrence of bradycardia by maintaining the HR at a higher baseline level, and to increase the safety of the surgery. Eventually, by stabilizing the HR, the operation ended smoothly.

In conclusion, with the improvement of surgical procedures and anesthesia methods, intraoperative TCR management is no longer intractable. However, recurrent intraoperative TCR poses new challenges. Anesthesiologists must be aware of this sudden physiological response, which may occur during various maxillofacial and brain surgeries. The cooperation of anesthesiologists and surgeons is mandatory and can save lives.

## Author contributions

**Conceptualization:** Yaping Wang.

**Data curation:** Qin Qin.

**Investigation:** Qin Qin

**Writing – original draft:** Qin Qin

**Writing – review & editing:** Yaping Wang
